# Total skin electron beam therapy

**DOI:** 10.3389/fonc.2025.1498855

**Published:** 2025-04-01

**Authors:** Lena Specht

**Affiliations:** Department of Oncology, Copenhagen University Hospital – Rigshospitalet, Copenhagen, Denmark

**Keywords:** total skin electron beam therapy, cutaneous lymphomas, mycosis fungoides, radiation therapy, hematological malignancies

## Abstract

Primary cutaneous lymphomas are highly radiosensitive. X-rays work well for localized cutaneous lymphomas. However, if disseminated in the skin and covering larger areas, as is commonly the case with the most common type, mycosis fungoides, x-ray therapy is not suited because the dose to underlying organs exceeds their tolerance. By contrast, electrons have a limited range of penetration, and are ideal for treating superficial lesions. Techniques have been developed to yield a fairly uniform dose to the entire skin surface and treating to a depth of about 1-1½ cm. Total skin electron beam therapy (TSEBT) is probably the most effective skin directed therapy for widespread primary cutaneous lymphomas. For many years the total dose used for mycosis fungoides was 30-36 Gy, given in small fractions. This treatment could only be repeated once. However, total doses of 10-12 Gy have now been shown to offer excellent response rates, and the treatment can be repeated up to 6 times, offering as much or probably even more palliation than the high-dose treatment. Today, most patients are treated with low-dose TSEBT, the higher doses reserved for patients with more resistant disease. Attempts have been made to use photon therapy for total skin irradiation, e.g., tomotherapy. However, even with the most meticulous of techniques there is too much dose in deeper structures, resulting in bone marrow toxicity even with low-dose treatment. This is never seen with electrons, even with high-dose therapy. Further research into optimizing TSEBT and exploring combinations with systemic treatments is ongoing.

## Introduction

Primary cutaneous lymphomas are the second most common primary extranodal lymphomas. The estimated annual incidence of primary cutaneous lymphomas (PCL) is 1/100,000 in Western countries. They must be distinguished from disseminated lymphomas with spread to the skin. Primary cutaneous lymphomas differ significantly from nodal lymphomas and from primary extranodal lymphomas in other locations in several important ways. They tend to remain localized to the skin for a long time, they have a much more indolent course and a much better prognosis than that of lymphomas of similar histological subtype in other locations, and they are treated differently (1–3). Hence, they are kept as separate disease entities in the histopathologic classification of lymphomas, both in the new WHO classification (4) and in the International Consensus Classification (5).

Primary cutaneous lymphomas are most commonly of T-cell origin, and the most common disease entity is mycosis fungoides and Sézary syndrome, a rare leukemic variant, which constitute 60-70% of all primary cutaneous lymphomas (1, 3). Most patients have widespread disease in the skin, but rarely dissemination to lymph nodes or internal organs, and even in these cases the major disease burden is often still in the skin. Except for rare cases of localized skin disease and rare cases of advanced cases treated with allogeneic stem cell transplantation, mycosis fungoides is incurable and treatment is generally palliative (1, 3, 6–8). These patients often live for many years, commonly decades, and the skin disease is often associated with distressing symptoms, e.g., itching, ulceration, and cosmetic problems. Hence, prevention and alleviation of these symptoms is of the greatest importance for maintaining a good quality of life. Skin directed therapies are the mainstay, for patients with early disease stages, patches or thin plaques, given alone, for patients with more advanced skin disease, infiltrated plaques or tumors, supplemented with systemic treatments (1, 3, 6–8).

## Radiation therapy for primary cutaneous lymphomas

Like other indolent lymphomas, mycosis fungoides is extremely radiosensitive (9). Doses of 8 Gy achieve complete response rates in > 90% (10). Indeed, mycosis fungoides was probably the first lymphoma reported to be treated with X-rays (11). Kilovolt X-ray therapy works well for small localized cutaneous lesions. However, if the disease is disseminated over larger skin areas, as is commonly the case with mycosis fungoides, it is not suited. The problem with X-rays, even kilovolt, is that if administered over large areas, the dose to the underlying internal organs exceeds their tolerance. By contrast, electrons have a limited range of penetration, limiting their effect to superficial tissues, the depth depending on the energy of the electrons.

The possibility of using artificially accelerated high-energy electrons in radiotherapy was anticipated by physicists in the 1920s (12, 13), but electrons with sufficient energy could not be generated at the time. In the 1940s physicists at the Massachusetts Institute of Technology in Boston constructed a Van de Graaff generator that was able to produce a 2.5 MeV electron beam. The generator was a huge construction, and still the maximum range of electrons in tissue was only around 10 mm (14). By this technology it became possible to treat large skin areas without damaging underlying critical structures (15).

**Figure 1:**
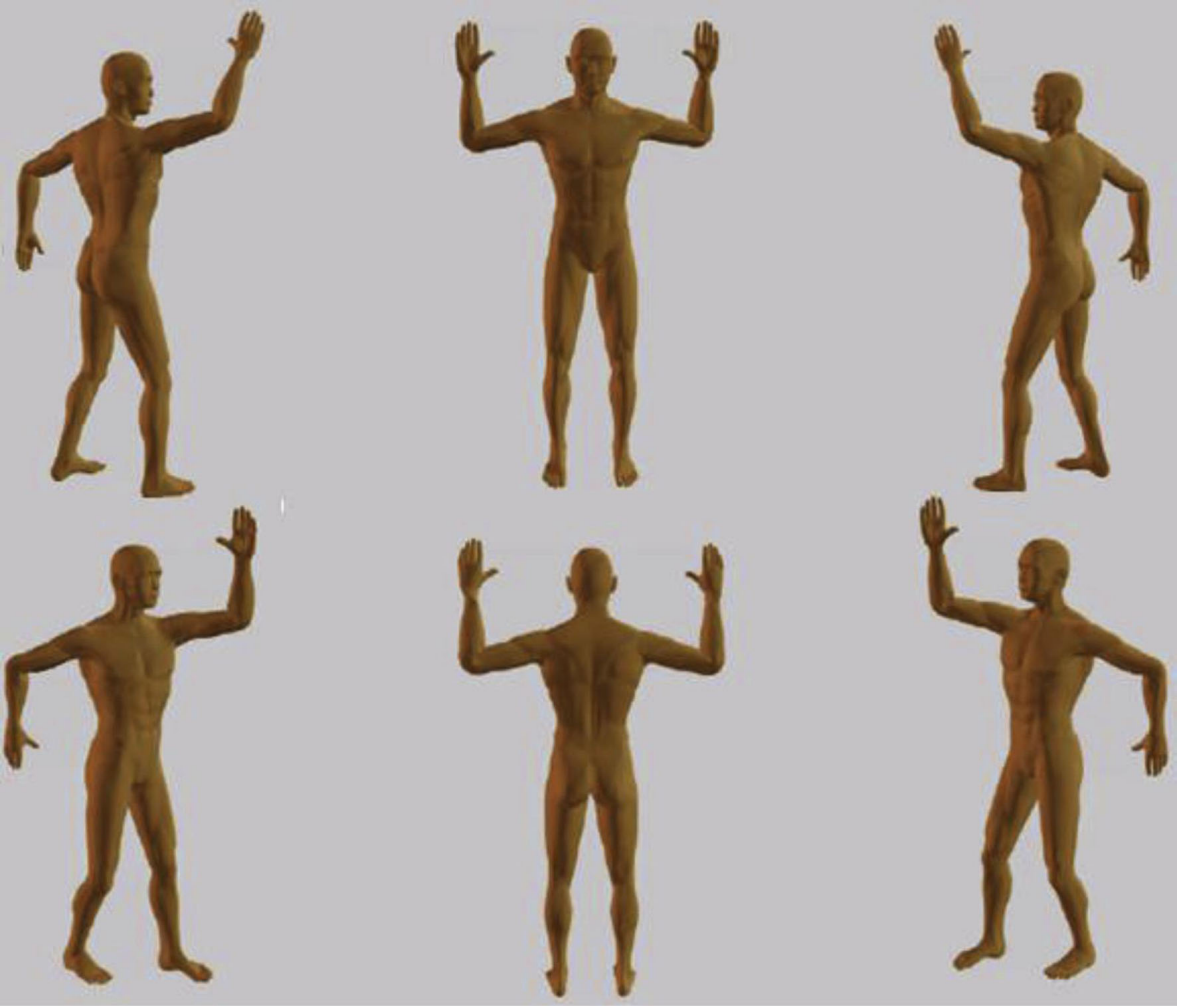
Patient positions for total skin electron beam therapy, 6-field technique. Reprinted from Specht et al., 2015 (9).

With the introduction of the linear accelerator, it became possible and practical to generate electrons for treatment with energies from 4 to over 20 MeV. Electron beam treatment of localized primary cutaneous lymphomas became and remains the optimal method for radiation therapy, enabling a homogenous dose to skin lesions with full skin dose and reaching the desired depth by judicious use of bolus. The challenge was to develop techniques for treating the whole skin in patients with disease disseminated in the skin, typically cases of mycosis fungoides (16). At Stanford a technique was developed where the patient was treated standing at an extended distance of 3-4 meters from the accelerator, with a dual field technique (angled from the horizontal position to be centered above and below the patient), and in 6 alternating patient positions (17, 18) (see **Figure 1**). This technique has become widely popular and is used in most centers offering total skin electron beam therapy (TSEBT). It provides a reasonably homogenous treatment of the whole skin (19–24), but supplementary electron fields must be administered to areas (e.g., scalp, perineum, soles) that are shielded during the TSEBT, and lead shielding of thin areas that would otherwise be overdosed (e.g., fingers and toes) should be applied halfway through the treatment. Rotational techniques, where the patient stands on a rotating platform during the treatment, have also been used, with the same need for supplementary electron fields to shielded areas and shielding of thin areas (25, 26). Translational techniques, where the patient is treated at a shorter distance lying alternately in the prone and supine position under the accelerator and being moved in the longitudinal direction are used less often (27, 28). The techniques for TSEBT are quite complex from a physical point of view, using the accelerator in a way that is different from the usual. Modern 3-dimensional treatment planning cannot be applied, and the techniques were developed by dosimetric measurements in phantoms and patients. The technical details were published in an AAPM (American Association of Physicists in Medicine) Report (29). The doses in different areas of the skin will vary depending on patient shape, anatomy, and position. Dose homogeneity is far from what we are normally used to in modern radiation therapy. There will be areas that are underdosed, but as the disease is incurable and the treatment therefore essentially palliative, this is acceptable. There will also be areas that are somewhat overdosed, but as the radiation doses needed in these diseases are fairly low, this is also acceptable. The technique can therefore also be used in other highly radiosensitive hematologic diseases, e.g., leukemia cutis (30). However, the technique is not suitable for solid tumors disseminated in the skin, which require higher doses.

Attempts have been made to use modern highly conformal X-ray radiation therapy for total skin irradiation. Helical tomotherapy with the patient in the supine position has been tested in several studies, consistently demonstrating significant hematological toxicity (31–34), which is never seen with TSEBT. Hence, even the most meticulous conformal X-ray technique is unable to limit the radiation to the skin to the same degree that can be achieved with electron beam therapy. TSEBT remains the recommended technique.

## High-dose TSEBT

When TSEBT was introduced in the 1960s a total dose of 30-36 Gy was generally prescribed for mycosis fungoides, often coined conventional dose TSEBT. This was based on studies showing that the complete remission rate was higher and the duration of remissions longer with this dose than with lower doses (35, 36). The skin is the largest organ in the body; hence the treated volume is nearly 20% of the total body weight. Treating such a large volume to this total dose was quite daring, and the treatment was given in small fractions, usually one fraction per day and with only four treatments per week. As the skin would in many cases become quite red about halfway through the treatment, a break of 1-2 weeks was usually made to avoid severe skin reactions. This made for a total treatment duration of 9-11 weeks. A recent meta-analysis of published data showed for patients treated with this conventional dose a complete response rate in early stage mycosis fungoides of 72% and an overall response rate of 100%, for advanced disease the complete response rate was lower (55%) but the overall response rate was similar (37). Patients will eventually progress, but the duration of the clinical benefit is clearly important, and a good measure of this benefit in mycosis fungoides is the time to next treatment (38). After conventional dose TSEBT it varies according to tumor burden and number or previous treatments, but may be up to a couple of years (35, 38). In most cases some form of maintenance treatment is given after TSEBT in order to maintain the response achieved with TSEBT for as long as possible (16) (see below). Patients with Sézary syndrome with blood involvement have generally not responded well to TSEBT, also because their skin is very thin and vulnerable and therefore does not tolerate irradiation so well, but data seem to indicate that TSEBT may be a useful adjuvant to systemic treatments in patients with significant blood involvement (39).

The toxicity of the treatment is actually quite manageable, rarely > Gr. 2 (37). Acute toxicities are erythema and dry desquamation, but hematologic toxicity is never seen with this treatment, because of the limited depth of penetration of the electrons. Temporary alopecia and loss of finger- and toenails is to be expected (around 70%), and patients are usually unable to sweat properly for 6-12 months. They may also (around 20%) experience variable limb edema during the first year after treatment. Second cancers in the skin, in particular squamous cell carcinomas, are seen with an increased frequency, but it is not possible to define the role of TSEBT in this as patients have always received many other carcinogenic skin directed therapies (40).

The main disadvantages of conventional dose TSEBT are that it takes a long time, which may be very inconvenient as most patients are fairly old and often have to travel to receive the treatment, and that it may be repeated only once in order to keep within the skin tolerance (41).

## Low-dose TSEBT

**Figure 2:**
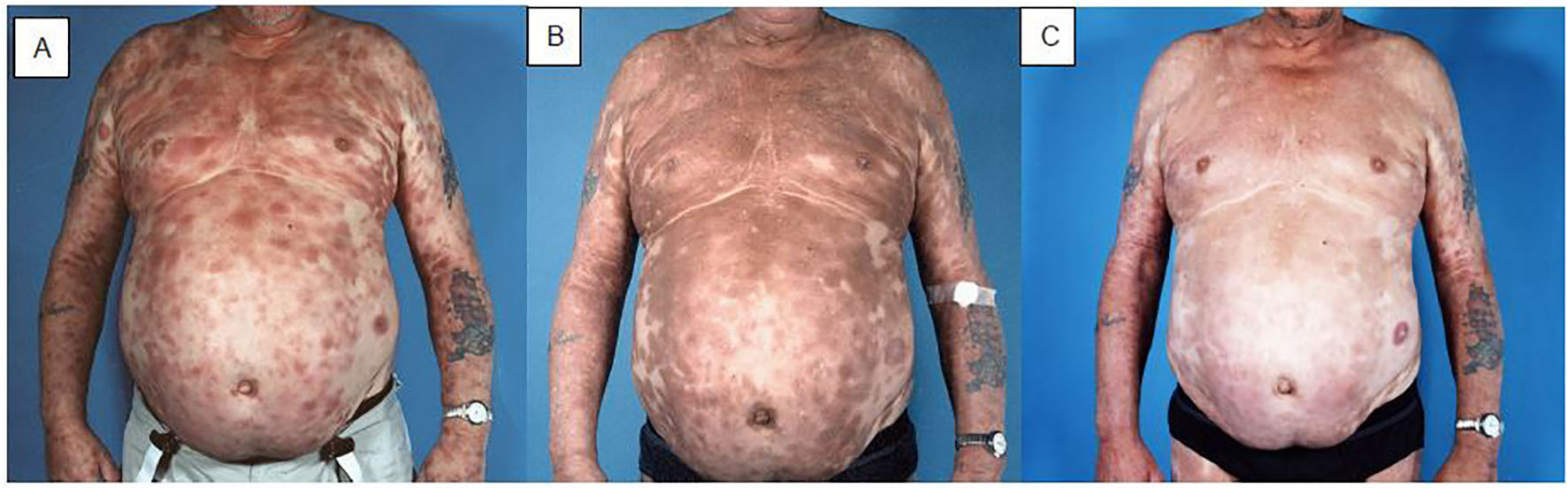
Patient with mycosis fungoides with disseminated plaques before **(A)**, one month after **(B)**, and one year after **(C)** TSEBT. During and in the weeks after TSEBT the structure of the skin normalizes, but there is still discolouration, which changes from red to brownish. The brown spots become paler and less prominent during the first year after treatment, but there will be some permanent discolouration.

With the realization that systemic indolent B-cell lymphomas are exquisitely radiosensitive with total doses as low as 4 Gy achieving durable local control in 70% of cases of follicular lymphoma and marginal zone lymphoma (42), we tested a similar dose with TSEBT for mycosis fungoides (43). The result was disappointing with a remission duration of only 2.4 months, confirming that T-cell lymphomas are less radiosensitive than B-cell lymphomas. We then tested a total dose of 10 Gy, 1/3 of our conventional dose TSEBT (44, 45). The response rate was 90%, with 70% complete or very good partial remissions. The median duration of remission was 5.2 months. Many subsequent studies using low-dose regimens of 10-12 Gy have confirmed these results (37, 46–57). The response rates with low-dose TSEBT were excellent. The duration of the responses was shorter than with the conventional dose, but the low-dose treatment could be repeated up to six times, offering as much or probably even more palliation than the conventional dose treatment with less toxicity and more convenience. Results from prospective trials of low dose TSEBT are shown in **Table 1**. Some form of maintenance treatment is usually given after TSEBT in order to make the response last for as long as possible (16, 58). Maintenance therapy is a highly individualized treatment, based on the experience with respect to response and tolerance with previous treatments in the individual patient. Maintenance therapy may be skin directed (16, 58–61), e.g., psoralen plus ultraviolet A (PUVA) and topical nitrogen mustard, or it may be systemic (16, 58, 61–63), e.g., interferon-alpha, retinoids, or newer drugs such as mogamulizumab, which is being tested in prospective trials. Combining low-dose TSEBT with systemic therapy may be effective also in patients with Sézary syndrome (64, 65). Low-dose TSEBT is today in general the preferred treatment, with higher doses reserved for more radioresistant cases. A typical case of mycosis fungoides with generalized plaques before and after TSEBT is shown in **Figure 2**.

**Table 1:** Prospective studies of Low Dose Total Skin Electron Therapy (TSEBT) for mycosis fungoides and Sezary syndrome.

Study	n	Dose and fractionation	Complete response rate (CR)	Overall response rate (OR)	Duration of response (DOR) (months)
Kamstrup, 2008 (43)	10	4 Gy/4 (4 per week)	20%	80%	2.7
Hoppe, 2015 (51)	33	12Gy/12 (4 per week)	27%	88%	16.3
Kamstrup, 2015 (45)	21	10 Gy/10 (4 per week)	29%	95%	5.8
Morris, 2017 (54)	103	12 Gy/8 (4 per week)	18%	87%	11.8
Song, 2020 (57)	25	12 Gy/6 (3 per week)	24%	88%	17.5
Georgakopoulos, 2020 (50)	8	12 Gy/6 (2 per week)	21%	92%	11.1
Elsayad, 2023 (67)	18	8 Gy/2 (1 per week)	17%	89%	12

Modified from Campbell et al. (16).

Early in the COVID pandemic, the International Lymphoma Radiation Oncology Group (ILROG) developed and published guidelines for use during the pandemic to minimize patients’ visits to hospitals when receiving radiation therapy for hematological malignancies (66). For TSEBT for mycosis fungoides a schedule of 4 Gy x 2-3 in weekly fractions was recommended, and has since been tested and found effective and well tolerated (67). Hypofractionation may offer advantages for patient living far from the treatment facility. However, each treatment takes quite a long time, which may be problematic for older patients.

## TSEBT as conditioning for allogeneic stem cell transplantation

At present the only treatment for advanced mycosis fungoides which has shown curative potential is non-myeloablative allogeneic stem cell transplantation, which induces a graft versus-lymphoma effect. A recent meta-analysis showed progression free survival at three years in one-third of patients, but with wide variation between studies (68). For a good outcome of this treatment the patient should be in as good a remission as possible before the transplantation. Conventional dose TSEBT is an effective debulking agent in the skin, and several studies have reported using this treatment as part of the conditioning regimen with promising results (69–71).

## Discussion

TSEBT is an effective and well tolerated treatment for mycosis fungoides and for other hematological malignancies when located in the skin. Electrons have superior physical characteristics which make them particularly suited for treating targets in the skin while sparing underlying normal structures. Hence, no other radiation therapy technique has been able to provide radiation therapy to the whole skin while avoiding toxicity from deeper-lying critical structures, in particular the bone marrow. TSEBT is a special treatment, it is delivered with varying techniques, all of them developed many years ago and by modern standards rather unprecise. Further research into optimization of this treatment will hopefully further improve outcome for patients with hematological malignancies in the skin.
